# Identifying misleading corporate narratives: The application of linguistic and qualitative methods to commercial determinants of health research

**DOI:** 10.1371/journal.pgph.0000379

**Published:** 2022-11-16

**Authors:** Iona Fitzpatrick, Adam Bertscher, Anna B. Gilmore

**Affiliations:** Tobacco Control Research Group, Department of Health, University of Bath, Claverton Down, Bath, United Kingdom; Universidad Austral de Chile, CHILE

## Abstract

Narratives are key to the way corporations represent themselves to the outside world, are important to the development of shared understandings and ultimately determine whether and how corporations are able to influence societal norms and participate in policy debates. A leaked corporate affairs strategy document, from the world’s largest transnational tobacco company, Philip Morris International (PMI), suggests a company concerned about credibility; it highlights “normalization” as a key strategic priority until at least 2024. This suggests that the PMI are seeking to rehabilitate their image and alter perceptions of their business. We designed a mixed-methods analysis of corporately authored content, combining quantitative querying of large bodies of text (Corpus Linguistics) with inductive coding of key themes to critically examine PMI’s corporate language and how these themes might impact public health debates. We systematically analysed a sample of PMI’s corporate communications (n = 170), comparing investor-facing (investor reports, slides and presentations as well as annual reports) and public-facing (YouTube content and Webpage content) communications covering a period of eight years (2012–2019). Our analysis identifies how PMI’s misleading external communication contradicts its core business focus and may threaten public health. In public-facing communications, PMI stress their commitment to transformation and change, while in investor-facing communications, they focus on cigarettes and reiterate the strength of their existing cigarette brand portfolios. This suggests that webpage and YouTube content provide a means through which PMI attempt to neutralise negative public perception of tobacco-product related harms and to present themselves as advocates of “better” consumer choice and even public health. The recurrence of transformation, sustainability, and science, as well as the co-option of united-nations terminology in their external-facing communications may serve to legitimise their involvement in policy arenas from which they currently excluded. We present a novel method through which corporate narratives can be monitored and critically assessed.

## Introduction

### Impact of tobacco control on the tobacco industry

Tobacco is uniquely harmful, and there has been a significant global response to its harms through the Framework Convention for Tobacco Control (FCTC). The FCTC, particularly Article 5.3, which aims to protect public health policy from “commercial and other vested interests of the tobacco industry”, has made it increasingly difficult for the tobacco industry to directly influence tobacco control policy. This and related tobacco control measures including extensive tobacco advertising, promotion and sponsorship (TAPS) bans and plain packaging regulations have increasingly denormalised the tobacco industry, reduced its ability to participate in public health debates and adversely affected cigarette sales particularly in higher income countries (HICs) [1]. However, country income continues to impact the effectiveness of tobacco control policies, with the least developed countries only legislating 45% of FCTC-recommended policies in 2014 (compared with 70% in HICs) [2].

PMI is the world’s largest transnational tobacco company [3]. In line with the trends in tobacco control detailed above, market research data show that the profitability of cigarettes began to decline globally in 2008 [4], with a marked decline in volume of cigarette sales of 20% between 2008 and 2018 [5]. It is in this context that PMI launched what it refers to as “smoke-free” products (a claim that has been contested [6]) and began making claims to be going “smokefree”. Though such products make up only a small share of the company’s total revenue (7.8% in 2019) [7], they have begun to form a key part of the company’s narrative.

Newer nicotine and tobacco products provide opportunities for image rehabilitation and leverage the public health community’s interest in “harm reduction”, which has been appropriated and misused by the tobacco industry [8]. The use of this language in their public relations and marketing initiatives enables the tobacco industry to align themselves with a “respected public health strategy” [9], and demonstrates attempts to “normalise” their presence in public health debates A leaked corporate affairs plan covering a period of 10 years (2014–2024) highlighted PMI’s concerns about its “demonization” and identifies “normalization” as a primary strategic priority, in which being “part of the solution” and “leverage[ing] NCD debate” is important [10]. Taken with the evidence of declining cigarette sales globally [4], this indicated that the company’s communications might help to unpack the ways in which PMI were hoping to achieve these ambitions.

### The role of corporate narratives in shaping public attitudes

The development of corporate narratives is a key way in which organisations manage business impressions and access new consumers [11]. Corporate narratives ultimately determine how corporations are able participate in public debates and influence the setting of shared agendas [12] as well as the development of shared understandings, including those related to public health [13] and tobacco control [14]. The power of corporations in the establishment of norms and the role they play in shaping the social, economic and political landscape is a well-documented phenomenon [15–17]. Given the discursive power of organisations and its role in the shaping of societal norms, there is an ever-present need to examine corporate language as an integral component of a profit-driven model “through which corporations propagate the non-communicable diseases pandemic” [18]. Our ability to address the commercial determinants of health depends on a clear understanding of the methods by which transnational corporations might seek to achieve influence and consolidate their political and economic power.

This paper therefore seeks to extend emerging methods on corporate narratives to the research fields of public health and commercial determinants of health. Using a novel method, we sought to identify patterns in PMI’s language, determine whether patterns in PMI’s language and communications varied across audiences and to assess the implications of those patterns for public health. This included understanding how these narratives might contribute to the “normalisation” of their business, their portrayal of tobacco-related diseases and their articulation of “the solution”.

## Material and methods

We developed a mixed-methods protocol blending methods and tools from corpus linguistics (CL) with inductive coding methods [19] beginning with an identification of audience types, as proposed by Breeze. We used Breeze’s model of corporate discourse to inform our methodology. That model outlines four key audience categories–“employees”, “investors”, “consumers” and a “wider audience” and was used to identify suitable units of sampling and analysis [20]. We selected “investors” and “a wider audience”, as the two categories that focus primarily on external audiences. We thus ensured the sample did not include communications exclusively meant for employees or other internal stakeholders and would be most relevant in building an understanding of narratives, which might include those related to normalisation.

**Fig 1:**
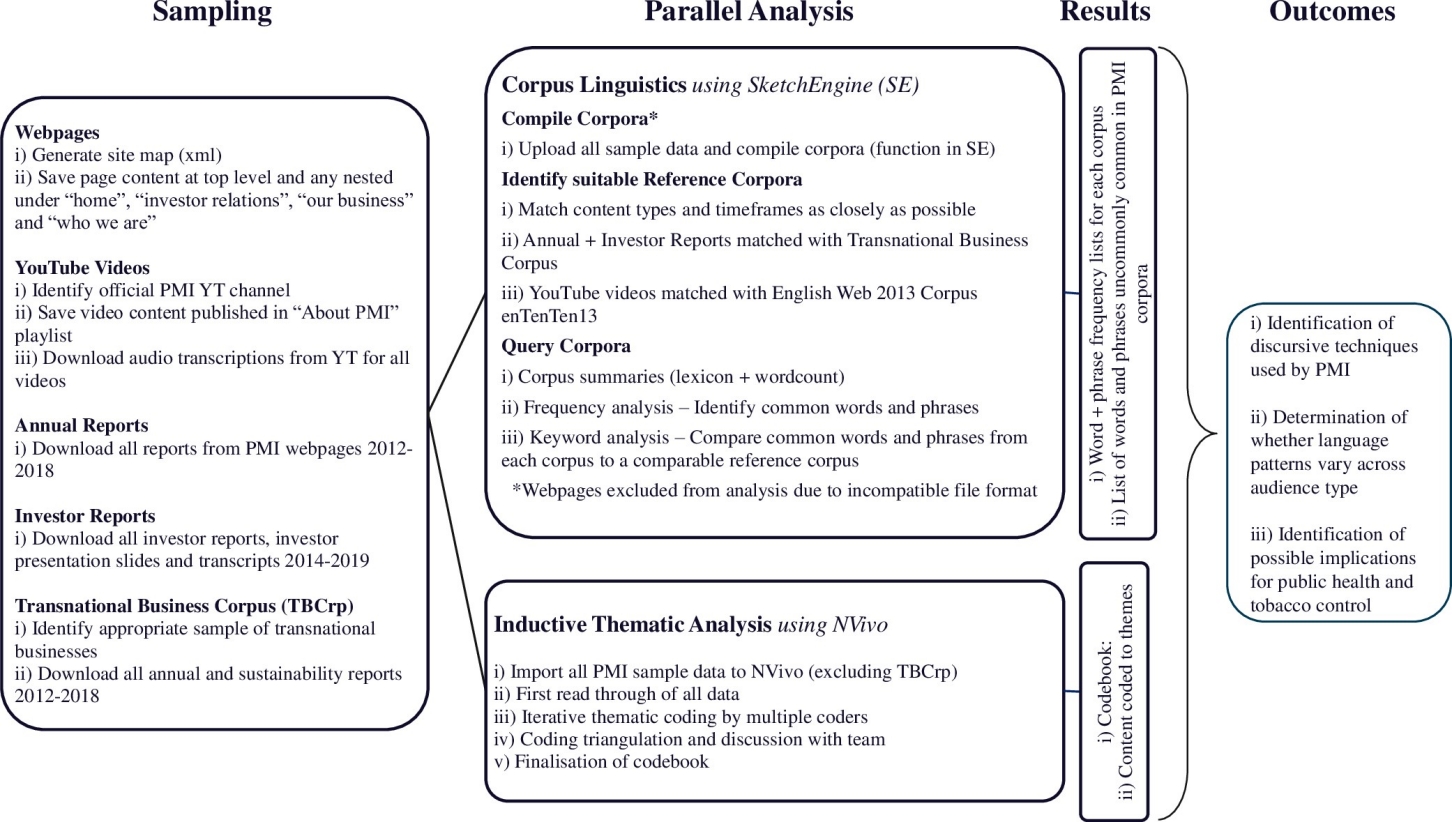
Method diagram.

Corpus linguistics uses large samples of "naturally-occurring" text to examine narrative and discourse [21]. Inductive coding is commonly used in qualitative research and was chosen to facilitate demonstration of links between findings and raw data [19]. We used the license-based textual analysis software SketchEngine [22] for compiling and querying custom bodies of text (corpora) and NVivo for inductive coding. The two methods were applied in parallel. A summary of the method, illustrating the sequence of the approach, and the steps taken at each stage is shown in Fig 1.

### Sample selection

For the “investor” targeted communications, we sampled annual and investor reports and presentations to shareholders, which included any associated slides and scripts. For the “wider audience” targeted communications, we sampled corporate webpages, and corporate YouTube videos. YouTube videos were selected for inclusion as YouTube is one of the most widely used online platforms worldwide [23] with a broad demographic engagement [24].

Annual reports, investor reports and presentations to shareholders were accessed and downloaded via PMI’s investor web pages. The sample of corporate webpages was downloaded manually according to a sampling filter developed specifically for this research. Corporate YouTube videos were accessed directly through PMI’s dedicated account. Automatically generated audio transcripts available from YouTube videos on a single playlist, titled “Inside Us” (n = 16) were saved in NVivo and checked for accuracy by IF, to allow for qualitative coding. Annual and investor reports were downloaded from PMI’s website [25].

### Webpage sampling

As there was no site map readily available through PMI’s website, we used a freely available online site mapping tool (xml-sitemaps.com) to generate an XML site map for www.pmi.com and sampled webpages according to the underlying logic of the page hierarchy. This sample consisted of pages at the “top” level (for example, www.pmi.com) and any pages nested under “home”, “investor relations”, “our business” or “who are we”. Target pages were captured using NVivo and Greenshot [26], and archived using WayBack Machine [27].

### Compiling corpora

For the corpus linguistic analysis, one sample corpus was compiled for each data type captured, except webpages (Table 1). Webpage content was saved as images (.jpg), so conversion to.txt files for analysis using SketchEngine, was not possible.

**Table 1:** Corpora compiled for CL analysis.

Corpus Name (+abbreviation)	Total word count	Lexicon (total unique words)	Date range covered
PMI annual report corpus	430,444	12,543	2012–2018
PMI investor reports corpus	574,556	18,549	2014–2019
PMI YouTube corpus	4,476	1,354	2019
Transnational corporations reference corpus	20,700,316	230,302	2012–2019

A large reference corpus, necessary for keyword analysis, was designed and compiled to match the language type and time frame covered by the sample corpora (2012–2018). It includes the 2012–2018 annual and sustainability reports from the top 40 of 100 top transnational companies ranked according to market capitalisation in US dollars in 2019 [28].

### Wordlists and keyword analysis

We conducted CL querying using SketchEngine, a specialist online resource for text-based research. “Wordlist” querying in SketchEngine reveals the most common words in the sample corpora. Keyword analysis reveals words that are “uncommonly common” in a selected corpus [29]. A standardised stoplist [30] (a list of words that are common to English, like ‘a’, ‘as’, ‘the’) was used to keep results relevant to the aims of the study. SketchEngine uses the following equation to generate a “keyness score”, indicating how likely a selected word or phrase is to appear in the selected corpus compared to a reference corpus.


$$\frac{{fpm}_{sample}+1}{{fpm}_{reference}+1}$$

Where fpm_sample_ is the normalized frequency (per million) of the word in the sample corpus, fpm_reference_ is the normalized frequency of the word in the reference corpus. + 1 is used as a smoothing parameter [31]. All reported keyness scores have been rounded to the nearest whole number. Frequency and keyness querying was non case-sensitive and we excluded non-words (numbers and symbols).

### Inductive thematic analysis

A purposive sample of the content was selected for coding (see Table 2). We used a general inductive approach, as outlined by Braun and Clarke [32] to identify recurrent themes [19]. The general inductive approach meant that codes were assigned following an initial read-through of all the data assigned to the primary coder. All sampled content was coded by a primary coder, with a sub-sample of that double coded by a secondary coder. Coding was discussed between primary and secondary coders and the final codebook agreed upon based on these discussions.

**Table 2:** Data capture and coding summary.

Content Type	Total sample		Subsample used for inductive coding (%)	
	Source count	Time period of publication	Coded by first coder	Coded by second coded
Webpages	55	2019	45	28
Annual Reports	7	2012–2018	100	14
Investor Reports	92	2014–2019	40	16
YouTube Videos	16	2019	100	28

The sample included annual and investor reports across several years in order to provide a more detailed picture of PMI’s language. Additionally, CL methods require a large word count, and the sampling of annual and investor reports over several years facilitated the accrual of a suitable sample size.

## Results

Frequency analysis of the text sample provided an overview of the language found in each type of communication. Using SketchEngine, we identified the most frequent words and phrases across content type (Table 3).

**Table 3:** Most common words in sample and reference corpora ranked in descending order of frequency (count).

PMI Annual Report Corpus	PMI Investor Report Corpus	PMI YouTube Corpus	Transnational Business Reference Corpus
Million (2515)	Excluding (5648)	People (27)	Financial (107,092)
December (2112)	Currency (5365)	Can (25)	Company (83,539)
Net (2008)	Total (4995)	Tobacco (24)	Million (75,206)
Share (1978)	PMI (4657)	Us (21)	assets (73,617)
Market (1963)	Net (4605)	Products (21)	Income (72,547)
PMI (1938)	Market (4493)	Will (19)	Net (68,069)
Tobacco (1851)	Income (4315)	PMI (18)	Value (65,944)
Total (1777)	Operating (4311)	One (17)	December (65,096)
Cigarette (1660)	Volume (4030)	Product (16)	Business (57,883)
Billion (1657)	Share (4018)	Change (16)	Total (56,167)

The word frequency analysis also revealed that the language of the YouTube audio differed from the annual and investor reports, which shared common vocabulary. The language of the YouTube videos included reference to change, products (non-specific) and people. Comparison with the transnational business corpus demonstrated the language used by PMI in their investor-facing communications does not differ from typical reports of the same type, across business sectors.

Word frequency analysis did not reveal anything unexpected, but helped to highlight the ways in which PMI’s focus in their communications aimed at a “wider audience” differed from the focus of their business-centred reporting.

The corpora overlap in varying ways; some phrases that were present in the YouTube videos were also used in the investor presentation slides, but did not appear in annual reports, for instance. The phrase "comprehensive action" for instance, was not present in the Annual Report Corpus but did appear in the annual meeting slides and the Year in Review YouTube videos. Comparatively, “sensory experience” was used in four of the annual reports (2012–2015) but did not feature in any investor reports, slides or scripts. "Unsmoke" did not appear in the Annual Report Corpus but was present in both the Investor Report and YouTube Corpora.

Frequency analysis highlighted the commonality of business terms in the annual and investor reports, as well as the prominence of tobacco and cigarette shipment volumes in PMI’s business reporting. PMI repeatedly identifies itself as a "leading international tobacco company" (n = 47) in their annual and investor reports. In contrast, the vocabulary of their YouTube videos contained less specialist business language, instead focussing on new product features such as “heating blade”.

Marlboro and IQOS were the only product brands to feature among the most common single words in the investor reports, with IQOS variably described as a “reduced-risk product”, “heated-tobacco alternative” and a “flagship smoke-free alternative”. In 2018, the investor reports begin to present IQOS as a “portfolio” rather than a single product.

The YouTube videos forefront a perceived struggle to participate in debates around tobacco control. This is often raised in tandem with mention of developing products, and “transformation”. PMI presents itself as a victim of exclusion being left out of debate by those unwilling to engage with them, while making efforts to do and be “better” (“offer better alternatives”). In a video titled “Open Mic with [redacted]” PMI’s representative expresses frustration at the exclusion “I mean, you know some people, whatever we say, whatever we do they always will try to connect it, that this is to promote our cigarettes”.

### Inductive coding

Inductive coding resulted in the identification of five key themes that were coded across all content types. Themes that did not appear across all content types were assigned to thematic codes nested underneath the top level. The five key themes identified were culture of permissibility, marketing, science (representation of), sustainability and transformation. Table 4 shows the breakdown of the final codebook and summarises the volume of content (in words) that was coded under each top-level code. Top-level codes aggregate the coding of their descendent codes.

**Table 4:** Volume of content coded to top 2 levels of the codebook.

Code	Description	Descendent codes	Volume of content coded (words)
**Culture of permissibility**	Content that involves the justification of corporate behaviour, includes moralising statements. Anything pertaining to the cultural acceptability of corporate activities, might include referencing commitment to non-corporate agendas (UN SDGs etc)	Total	17,073
**Marketing**		Total	1,274
	Content that mentions marketing campaigns or brands. Include use of promotional language, and use of hashtags.	#unlabel	44
		#unlimit	30
		2.0 architechture	948
		Be marlboro	32
		Don’t be a maybe	98
**Science (representation of)**		Total	3,948
	Content mentioning science or technology, or related concepts, with a scientific focus. Includes science, laboratory research, mention of scientific/technical staff	Laboratory/depictions/equipment	67
		Other Technology	308
		Research/research studies	1821
		Science and technology	2790
**Sustainability**		Total	3,758
	Content focussed on sustainability or related activity or ambition. Includes references to UN SDGs, CSR, environmental sustainability etc	Agriculture OR labour	969
		Environmental	1833
		Equality and Diversity	169
		Health OR wellbeing	261
		Sustainability “Other”	295
		Sustainability None-specific	133
**Transformation**		Total	15,949
	Any reference to change, alteration, historical difference. Any use of language associated with radical change (transformation, innovation etc). Include mentions of future endeavours and any invocation of an altered historical state esp “the future”	Cessation OR quitting	39
		Disruption OR change OR innovation	7168
		Growth/expansion	5281
		Provenance/Heritage	349
		“Smoke-free”	1924
		The future	1299
		Unsmoke	445

Transformation was the most frequently coded topic across both the YouTube videos and across all of the website pages. Transformation was largely mentioned in reference to the development of a “new future” or a "smoke-free future". This connection is reflected in the code description in the final codebook (see descriptions in Table 5).

**Table 5:** Examples of content coded to top levels in the codebook.

Code	Example content	Content source
**Culture of permissibility**	• That’s really what we wanna be part of regular, smart people’s lives and we wanna do it in these kinds of forums and dammit, we are not apologising for being Philip Morris International, we’re doing the best we can, which is offering better for you	**PMI at Cannes Lions YouTube Transcript**
	• The integrated efforts of our contributions, sustainable tobacco production programs and labor initiatives serve to improve the livelihoods of communities around the world	**2013 Annual Report**
	• Further to informed consumer choice, appropriate regulation would provide clarity for manufacturers and would advance the public health agenda	**2015 Annual meeting of Shareholders script**
**Marketing**	• We developed the new “Don’t Be A Maybe–Be Marlboro” campaign, which was initially implemented in Germany and rolled out to approximately 20 markets in 2012. With the new campaign, Marlboro encourages adult smokers to be decisive, trust themselves and follow their inspiration.	**PMI 2012 Annual Report**
	• Sometimes we form opinions of others without much thought. Let’s stop and ACT to #UNLABEL	**PMI Year in Review 2016 YouTube Transcript.**
	• We are very pleased with the performance of our flagship brand. Last year, we launched Marlboro 2.0, focusing primarily on European markets.	**Annual meeting of shareholders script 2015**
**Science (representation of)**	• science and technology and innovation are at the absolute heart and core of everything we’re doing to transform our company and transform the lives of men and women who smoke all across the world	**PMI at Cannes Lions 2018 YouTube transcript**
	• Our scientific assessment program, outlined at www.pmiscience.com, made substantial progress last year across all four RRP platforms.	**PMI Annual report 2018**
	• we have put in place a rigorous and transparent scientific pre- and post-market assessment program that is unmatched in the industry. This program has become a cornerstone of our external engagement, in which we advocate for public health authorities and policy makers to adopt the same evidence-based approach to designing regulations that foster healthier consumer behavior compared to continued smoking.	**Annual Meeting of shareholders script 2019**
**Sustainability**	• We support programs that help make a difference in communities where our employees live and work, as well as in farming communities where we source tobacco.	**2012 Annual report**
	• Our 2018 Annual Report reviews how we have refined our sustainability strategy to align with societal expectations.	**2019 proxy statement**
	• Last year, the adoption of the United Nations Sustainable Development Goals and the Paris Climate Conference defined a global agenda for a better world, and urged civil society, business and government to work together towards achieving the established targets.	**2016 annual meeting of shareholders script**
**Transformation**	• iQOS and our other RRPs constitute our single-largest growth opportunity.	**PMI Annual report 2015**
	• we have a vision of what things are going to be like as technologies advance, as we become more committed to a healthful, yeah, a healthful vision of the future	**PMI at Cannes Lions YouTube Transcript**
	• The foundation of RRP commercialization efforts is PMI’s “innovation machine,” which includes unparalleled expertise in critical areas, such as technology, engineering, product development, quality assurance, scientific substantiation, design and user experience, brand retail, human insights and behavioral research, digital solutions and experience marketing, and responsible business practices.	**PMI Annual report 2018**

Their commitment to doing and being “better” was largely coded under “culture of permissibility”. This included emphasis on the importance of PMI in the development of effective and proportional solutions to global issues, including tobacco control. The claimed exclusion of PMI from certain platforms and conversations was a recurring theme in their YouTube content.

## Discussion

This paper presents a mixed-methods approach to the investigation of corporate communications, and presents examples of the application of the method to explore the language and discursive themes present in the external communications of PMI. By combining large-scale linguistic analysis with in-depth iterative thematic coding, the method has illuminated the main narratives being voiced in each type of communication.

Both the vocabulary used (common and key words and phrases) and the themes identified have illustrated that PMI’s communications with a “wider audience” (YouTube videos and webpage content) differs to their communications with “investors” (annual and investor reports). The former feature discussions of progress and change and while the latter focus on the operation of the business and illustrate, in detail, the key role that cigarettes and other tobacco-based oral products play in their business. In addition to language patterns and key themes, the analysis also revealed several related discursive techniques that echo the findings of previous work into corporate communications and their importance in advancing narratives that counterbalance perceived industry harms [33–38].

Several techniques of neutralisation were evident in the coded material. Our findings are broadly consistent with several papers dealing with corporate action and corporate accountability; Fooks et al [33], Holzer [34], Whyte [35] and Piquero [37], show that corporations are able use the “structurally dominant position they enjoy” in order to shape public understandings of their business [35]. Our analysis suggests that PMI is seeking to normalise their participation in public health discussions while relying on their portfolio of conventional combustibles to fulfil obligations to shareholders. They achieve this through alignment to already-sanctioned public health discourse (the UN’s SDGs), the condemnation of the tobacco control community [33, 35, 37, 38] and by shifting accountability onto consumers.

### Alignment to already-sanctioned discourse

Corporations leverage their involvement in non-business activities to demonstrate their commitment to a productive society and taking an interest in issues salient to the public in order to improve their image [33]. This allows them to cast themselves as legitimate advocates for change, a tactic which can provide access to governments and policy makers [33]. PMI is no different. By co-opting familiar terms like the UN’s SDGs, and common terms like “transformation”, “science” and “common sense”, PMI are able to manipulate how audiences come to understand these terms, thereby shifting consumer expectations, and ultimately, controlling the limits of “acceptable” corporate behaviour.

The sample of communications discussed here suggest that PMI is seeking to shape perceptions about the essential role of tobacco corporations in promoting public health and contributing to sustainable development. Their co-opting of UN vocabulary, and in the case of the 2015 annual report, imagery taken directly from the UN’s SDG model, helps to obfuscate the reality of their business, that is as a "leading international tobacco company", where cigarettes and other combustible products continue to account for more than 80% of their net revenues [39] causing huge harm to economies, environment and development, as well as of course health [40–43].

Furthermore, the recurrence of “common sense” and the prominence of rationality in their corporate storytelling highlights PMI’s efforts to establish new understandings of what is "better", and what is “serious”. Our analysis suggests that PMI is seeking to redefine norms associated with public health agenda setting. Their emphasis on value-laden statements about consumer choice and common sense is concerning. Corporations like PMI play a “hegemonic role…in shaping ‘common sense’ understandings of the world” [35] and should be carefully critiqued, particularly when those corporations are communicating directly with consumers or participating in public health agenda setting and regulation. David Whyte points out that “the successful dissemination of common sense is crucial to elites in providing popular consent for particular strategies of rule” [35]. In the case of PMI, these “strategies of rule” might include the involvement of tobacco companies in policy making, less stringent tobacco control policies, and an insistence on the apparent irrationality of the pursuit of a market without conventional tobacco products.

### Condemning the tobacco control community

The prominent narrative of PMI’s exclusion from conversations about tobacco control is striking. This narrative is a variant of the well-documented technique of neutralisation where corporate actors claim “victimisation by state regulation” and emphasise the “punitive intervention” of regulatory bodies [35] over their own responsibility.

Whilst suggesting their exclusion from discussions concerning tobacco and tobacco control is baseless, PMI is working to discredit those who oppose the tobacco industry (“I mean, but let’s be serious about this whole thing” [44], framing them as trivial and unserious while stressing PMI as an important agent in the development of solutions. They repeatedly stress the importance of their own involvement “in order to make any ultimate change, you’ve actually got to speak to the people that maybe you feel uncomfortable speaking to because those are the ones that are going to actually make that change happen” [45]. Those who oppose the tobacco industry are not unwilling, but required to ensure that tobacco control related policy setting is protected from the tobacco industry (Article 5.3 of the FCTC). The tobacco industry’s exclusion from these discussions is based on extensive evidence that they have for many years intentionally and detrimentally interfered in public health policy making [46] related to tobacco control.

### Shifting accountability onto consumers

PMI repeatedly stresses the importance of consumer desire in driving the market for new tobacco products. in the YouTube video “The Power of Truth” [45], consumer choice is connected to the broader right to personal freedoms and freedom of choice–the video states, “you can’t just say don’t”. The YouTube transcripts and website pages clarify PMI’s focus on personal and creative freedom, including consumer choice. The emphasis on individuals who “would otherwise continue to smoke” is mentioned in annual and investor reports, where the company’s RRP portfolio is framed as “the most promising path to providing a better consumer choice” [47] is misleading. It shifts focus away from the corporation and is a misrepresentation of the hugely addictive nature of tobacco. Choice is not, as PMI suggests, the crux of the issue. Existing research suggests the opposite is true–around 90% of smokers (in the US, Canada, Australia and the UK) want to quit smoking altogether and express regret at ever having started [48]. The emphasis on consumer action further facilitates the normalisation of corporate involvement in public policy agenda setting, in so far as they can use these narratives to carefully define the limits of their own accountability [34].

### Strengths and limitations

To our knowledge this is the first time this approach has been applied to public health. Our method provides a broadly applicable and practical framework for the sustained and systematic monitoring of the narratives of “big business”. We have also compiled a large-scale corpus of business language that can be used by other researchers. By combining corpus linguistics approaches with the use of inductive coding, we were able to effectively manage the analysis of a huge volume of data whilst uncovering key ideas through qualitative analysis. Visual content from YouTube content was not systematically analysed due to resource constraints; further research seeking to use audio-visual content should make full use of the data types available. The software we used to conduct our analysis (SketchEngine) is subscription-based, but there are several similar CL software packages that are open-source, so funding need not be a limitation to future application of this method. Similarly, although inductive coding is time-intensive and required some training, it can be easily employed without prohibitive cost.

### Public health implications

By understanding the “system of statements” in PMIs communications, we have to unpack the various constructions of meaning that are specific to PMI [49]. The results presented here highlight striking parallels with previous analyses of corporate content, which highlight the use of several “techniques of neutralization” as devices to alter perceptions as well as important political tools in the legitimisation of corporate behaviour and the minimization of corporate responsibility for tobacco-related harms.

Our analysis shows PMI is using its public facing corporate communications as key platforms to neutralise its documented misconduct and harms. Its narratives seek to legitimise the company whilst transferring culpability for adverse health outcomes and lack of progress in reducing tobacco-related harms onto consumers and the public health community, despite overwhelming evidence the tobacco industry has been the single largest barrier to progress [1, 50–52]. PMI’s story-telling distracts from the specificities of its business while promoting non-specific visions of transformation seeking ultimately to enable the company to re-enter the policy arena. Our findings signal this is a disingenuous narrative–one provided publicly, with a quite different narrative presented to investors. They are entirely consistent with a company acutely aware of its “demonization” and the need to urgently renormalise its image. The similarity of these findings to analyses of the tobacco industry’s conduct in the 1990s [53–56] signals that little has changed. Our findings mirror those of earlier researchers, who have shown than the tobacco industry employs tactics aimed at altering cultural perceptions of itself (the company) and its products [56, 57].

PMI may have newer nicotine and tobacco products which could, with appropriate regulation, contribute to reducing the appalling harms from tobacco, but such products, like heated tobacco products, are currently leveraged by the tobacco industry in order to undermine existing regulatory efforts and progress in tobacco control [58]. Its primary interests–maximising profits from both conventional and newer tobacco products—remain fundamentally opposed to those of public health. A clear distinction must therefore be made between a product and a producer: a product may play a role in tobacco control; that does not mean the producer of that product can. As such, Article 5.3 of the FCTC which stipulates that tobacco control public health policies should be protected from the “commercial and other vested interests of the tobacco industry” [59] remains more important than ever.
